# A Two-Phase Approach for Conditional Floating-Point Verification

**DOI:** 10.1007/978-3-030-72013-1_3

**Published:** 2021-02-26

**Authors:** Debasmita Lohar, Clothilde Jeangoudoux, Joshua Sobel, Eva Darulova, Maria Christakis

**Affiliations:** 8 Eindhoven University of Technology, Eindhoven, The Netherlands; 9 Aalborg University, Aalborg East, Denmark; 10 MPI-SWS, Saarland Informatics Campus, Saarbrücken and Kaiserslautern, Germany; 11 University of Rochester, Rochester, USA

## Abstract

Tools that automatically prove the absence or detect the presence of large floating-point roundoff errors or the special values NaN and Infinity greatly help developers to reason about the unintuitive nature of floating-point arithmetic. We show that state-of-the-art tools, however, support or provide non-trivial results only for relatively short programs. We propose a framework for combining different static and dynamic analyses that allows to increase their reach beyond what they can do individually. Furthermore, we show how adaptations of existing dynamic and static techniques effectively trade some soundness guarantees for increased scalability, providing conditional verification of floating-point kernels in realistic programs.

## 1 Introduction

Floating-point arithmetic is widely used across many domains, including machine learning, scientific computing, embedded systems, and the Internet of Things. Floating-point computations resemble real-valued arithmetic, but provide only finite precision, which commits roundoff errors at potentially every operation. While these errors are individually small, they propagate through an application and can make its results meaningless [47]. In addition, floating-point arithmetic features special values such as not-a-number (NaN) and Infinity [48]. As a result, these computations are very challenging for developers to reason about and debug manually. There is, therefore, a clear need for automated verification and debugging techniques for such computations.

Unfortunately, today's techniques do not handle realistic floating-point programs well. Consider for example a program that simulates the interaction of several bodies under gravity. We took a C implementation of this N-body problem from Rosetta Code [5], which takes as input the masses, positions and velocities of—in our case—three bodies, and shows their evolution over a number of time-steps. The entire program is moderately-sized with 108 lines of code. Suppose that we want to verify the absence or presence of special floating values and cancellation (i.e. large roundoff) errors in this program. None of the currently available floating-point analysis tools is able to do this.

```
1  int main(int argc, char* argv[]) {... // Reads masses, positions and velocities
2    for(int i=0; i<timeSteps; i++) { simulate(mass, pos, v); ...}
3  }
4  void simulate() { compute_accelerations(mass, pos); ...}
5  void compute_accelerations(double mass[], vector pos[]){
6    for(int i=0;i<bodies;i++){ ...
7      for(int j=0;j<bodies;j++) {if(i!=j) {
8        acc[i] = numerical_kernel(mass[j], pos[i], pos[j], acc[i]);}}}}
9  vector numerical_kernel(double mass, vector pos_i, vector pos_j, vector acc) {
10   return addVectors(acc, scaleVector(g*mass/pow(mod(subtractVectors(pos_i,pos_j)),3),
         subtractVectors(pos_j,pos_i))); // compute acceleration
11 }
```

**Listing 1.1.** Snippet of Rosetta code N-body simulation

State-of-the-art static roundoff-error analysis tools [33,31,30,60,65,72] are in principle capable of proving the absence of both special values and large roundoff errors by computing an abstraction of the possible behaviors. However, they work only on small programs, mostly consisting of a single function, and thus do not work for our N-body example. The static tools that do scale [11,63,43] suffer from large over-approximations due to abstractions and thus effectively cannot prove the absence of issues either. Bounded model checking [52] or SMT decision procedures [25] perform exact bit-precise reasoning, but do not scale enough due to the complexity of floating-point arithmetic.

On the other hand, there exist dynamic analyses that search for concrete inputs proving the presence of Infinities [38], NaNs or cancellation errors [10,21,78]. We could not apply any of these tools on our example, to a large part because they, too, have been designed for relatively small programs. More guided techniques such as symbolic execution [57] rely on a back-end SMT solver, for which floating-point theories have very limited scalability.

We evaluated representative available tools on a new collection of floating-point benchmarks and get similar results for most of them (Section 5).

We observed that often only a relatively small part of a program performs complex numerical computations—we call these parts the *numerical kernels*. Existing state-of-the-art floating-point analyzers can be applied to these kernels, provided that one can supply a precondition that bounds the kernel's input ranges (their minimum and maximum values). Obtaining such preconditions manually is challenging, since the kernels are usually nested in loops as functions. Listing 1.1 shows a subset of the N-body example; the numerical kernel that we identified is on line 9, nested behind several for-loops and function calls.

Based on this observation, we propose a two-phase analysis that combines different program analyses to conditionally verify the absence of special values and cancellation errors in numerical kernels 'concealed' in large programs. First, we employ a scalable program analysis to infer the ranges of a kernel's inputs in the context of the containing application. In the second phase a different program analysis assumes these ranges to verify the kernels.

The main insight behind this combination is that the first scalable analysis does not need to perform sophisticated floating-point reasoning; the domain specifications required for the second numerical analysis need to only capture input ranges of variables.

The main challenge in our two-phase analysis is the first phase where our objective is to infer the ranges of the kernel inputs automatically. We first attempt to verify the numerical kernels fully soundly. Hence, we utilize abstract interpretation to infer sound ranges of kernel inputs. In case it is unable to infer useful (finite) ranges for the kernels, we propose to adapt existing blackbox and greybox fuzzing techniques [12], and evaluate them in their ability to produce large kernel input ranges capturing as many feasible inputs as possible.

After inferring the kernel ranges, the second phase utilizes a slightly adapted existing static and sound roundoff error analysis [30] to verify the kernels. In case this analysis produces warnings for special values, we additionally utilize SMT-based bounded model-checking [52] to check for spurious warnings.

Although there is a large body of work on combining different program analyses, our goal of analyzing real-world applications to verify their numerical kernels is novel. Our combination is specifically tailored to this setting, by considering the intricacies of floating-point arithmetic and the limitations of today's analysis techniques in reasoning about them.

Using a dynamic analysis in the first phase means that we are only able to infer approximations of the kernel input ranges. Consequently, we can verify the kernels only *conditionally*, because the verification is performed under the assumption that the input-domain specifications precisely describe possible values of the kernel inputs. Thus, we take a practical standpoint and relax the soundness guarantees in favor of wider applicability of today's static floating-point roundoff-error verification techniques.

Our evaluation shows that for 16 out of 24 kernels, this approach is able to verify that no special floating-point values occur; for 3 of those kernels, verification is sound. For 14 kernels, we additionally show the absence of cancellation errors that are a main cause of large roundoff errors.

*Contributions* To summarize, our paper makes the following contributions:

a) a two-phase framework that combines dynamic and static analyses to conditionally verify the absence of floating-point special values and large roundoff errors in kernels,
b) a novel guided blackbox fuzzing technique to infer kernel ranges, implemented in an open-source prototype tool called Blossom, and
c) an evaluation on a new benchmark set of mid-size numerical programs.

Our benchmarks, the tool Blossom as well as scripts of all of our experiments are available at `https://github.com/dlohar/blossom`.

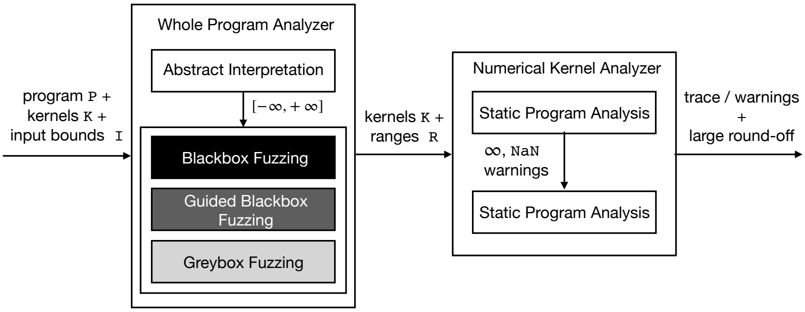

**Fig. 1.** Overview of our approach

## 2 A Two-Phase Approach

Figure 1 shows an overview of our two-phase approach that strives to increase the reach of existing floating-point analyses of floating-point numerical kernels. Our key observation is that such kernels appear in real-world applications from a variety of domains, but they are often 'hidden' behind several function calls and other non-numerical code that the round-off analyzers cannot handle. The first phase infers bounds on the input variables of a set of numerical kernels $\mathcal{K}$ that have been identified by a user in a program $\mathcal{P}$. In the second phase, we utilize these ranges to (conditionally) verify the kernels, i.e. to (conditionally) prove the absence of special values and large roundoff errors.

An alternative strategy would be to identify the largest kernel input ranges for which correctness can be guaranteed. However, even if one could infer such preconditions (we are not aware of a tool that performs such a backward analysis), our techniques for the first phase would still be needed to determine whether the program can execute the kernels on inputs outside of the safe ranges.

### 2.1 First Phase: Whole Program Analysis

In the first phase we have a whole program analyzer that, starting from the *program inputs* constrained by $\mathcal{I}$, infers bounds $\mathcal{R}$ on the *kernel inputs* automatically. These bounds are crucial, as the presence of cancellations and special values directly depends on the ranges of possible values; an unbounded input range will, in general, also lead to unbounded roundoff errors and special values.

To obtain the kernel ranges, we need to analyze the entire program. In general, it is infeasible to compute the exact ranges, so that we want to approximate them. We propose to first use a sound static analysis, which computes an over-approximation of the true ranges. They thus cover all feasible inputs, but additionally also spurious ones, so we want these ranges to be as tight (small) as possible. If the abstractions necessarily performed by the static analyzer become prohibitively large, we propose to use dynamic analysis to compute an unsound approximation of the kernel ranges. These ranges should be as wide as possible to capture as many concrete executions as possible.

*Sound Static Analysis* We choose abstract interpretation [26] and specifically the industry-strength analyzer Astrée [63] to infer a sound over-approximation of the kernel ranges, as Astrée scales for large programs with complex code and data structures and comes with a variety of abstract domains.

The choice of the abstract domain in Astrée is, in general, a trade-off between the amount of over-approximation and the analysis running time. The interval domain abstracts a set of concrete variable values by their lower and upper bounds: $[\underline{x}, \overline{x}] := \{x \mid \underline{x} \leq x \leq \overline{x}\}$. While operations on interval arithmetic [64] are efficient, intervals cannot capture correlations between variables and therefore over-approximate the real behavior (e.g. $x - x \neq 0$ in interval arithmetic). Nonetheless, for our benchmarks we have not observed any noticeable difference in the results with more sophisticated domains (e.g. octagon). This is likely due to our benchmarks having many nonlinear operations. Hence, we choose the interval domain as the numerical abstract domain for our purpose.

*Dynamic Analysis* Fuzzing finds inputs that demonstrate certain (unwanted) behavior. We propose to fuzz a program and at the same time monitor the kernel inputs to record the lower and upper bounds seen during concrete executions.

We instrument each user-specified kernel in the program with a kernel monitor that keeps track of the smallest and largest value seen for each kernel input. We repeatedly execute the instrumented program and report the minimum and maximum values seen for each kernel input over all executions. This approach crucially depends on the choice of program inputs that are used for fuzzing. We propose and experimentally compare blackbox, guided blackbox, and directed greybox fuzzing [12] as methods for input selection in Section 6.

*Blackbox* fuzzing is a naive but effective technique in many testing situations. In our setting, the blackbox fuzzer randomly draws inputs from the program ranges $\mathcal{I}$, i.e. without any reference to the internal structure of the program.

We further propose *guided blackbox* fuzzing that is guided toward enlarging the kernel input ranges. For this, the program input generator records those inputs that have widened the kernel ranges, and randomly generates new inputs that are within a certain (small) distance from these, in the hope that the new inputs would enlarge the monitored ranges even further.

While blackbox techniques are straightforward to implement, they do not take into account the program structure. We thus evaluate an adaptation of *directed greybox* fuzzing, implemented in the the state-of-the-art tool AFLGo [12] that can be directed toward specific program locations, while exploring as many different paths in the program as possible. We first fuzz the program to obtain an initial estimate for the kernel input ranges with AFLGo (targeting the kernel). Then, we employ AFLGo in a refinement loop that iteratively attempts to widen the currently seen kernel input ranges. We instrument the kernels with conditional statements that check whether a kernel input is outside of the current kernel range. We use this conditional statement as a target for AFLGo, effectively directing it to find kernel inputs that are outside of the current estimate. If AFLGo finds a program input that widens the current kernel input range, we update it accordingly and iterate the process until a user-defined timeout.

### 2.2 Second Phase: Numerical Kernel Analysis

With the ranges ($\mathcal{R}$) inferred in the first phase, we analyze the user-identified numerical kernels ($\mathcal{K}$) in the second phase with a static analyzer. Our objective in the second phase is to either show the absence of special floating-point values and large roundoff errors in a kernel or to generate warnings for the potential presence of such values.

We use the sound floating-point roundoff analysis tool Daisy [30], which automatically proves the absence of special values and computes an absolute error bound for each kernel output. When Daisy generates a warning that special values can potentially occur, we use a SAT/SMT-based model checker that performs exact floating-point reasoning and that can identify spurious warnings.

By itself, the error bound on the *kernel* output is not particularly helpful, however, since we do not know how this error propagates to the *end* of the program (although there exist scalable analyses that potentially can compute this information, e.g. [61]). That said, for many numerical applications the exact error bound is not important, since the algorithm itself is already approximate. For these applications, it is thus sufficient if we can show that the *roundoff errors are not too large*. We thus modify Daisy to report a warning when it detects a possible *cancellation*, i.e. when an arithmetic operation increases the relative error significantly (e.g. when two values that are close in magnitude get subtracted [42]). Additionally, Daisy includes an optimization procedure that can improve the accuracy of the kernels by rewriting the arithmetic expressions to commit smaller roundoff errors. We provide more details in Section 4.

### 2.3 Soundness Guarantees

To summarize, using the extended Daisy analysis, we can conditionally verify that kernels do not result in any NaN or Infinity, and that they do not commit cancellation errors, i.e. lead to large roundoff errors. When the kernel input ranges are computed soundly using abstract interpretation (e.g. Astrée), our verification is conditional in that we only verify the absence of cancellations for the kernels, but not for the rest of the program.

When the ranges are computed using dynamic analysis in the first phase, they include more concrete values than the fuzzer witnessed. Values between the lower and upper bound are not necessarily observed by the fuzzer, and are also not necessarily feasible. If one were to consider only values witnessed at runtime, then it would be possible to analyze kernels for individual traces, although this would be quite expensive [10]. However, if we can soundly show that no special values or large roundoff errors (cancellations) occur inside a kernel for a given input range, we have shown this for more executions than can be explored by dynamic testing in general (since there are usually too many floating-point values to explore exhaustively). Unlike for a NaN or Infinity that are obvious to detect, cancellation cannot, in general, be detected by inspecting the computed results and thus our combination is valuable.

## 3 First Phase: Whole Program Analysis

*Abstract Interpretation with Astrée* We utilize Astrée as it scales for large C programs with complex code and data structures. We add wrapper functions to provide bounds for global variables, since Astrée does not assume ranges for global variables directly. We further annotate the kernels $\mathcal{K}$ with Astrée's `__ASTREE_log_vars()` construct. This construct records the range information that Astrée logs about the kernel inputs at the entry of the kernels.

Note that the analysis of Astrée can be extensively parameterized with the knowledge of the program under analysis. Although this makes the analysis even more precise, it requires vast manual effort and knowledge of the intricacies of the program. To avoid this, we parameterize Astrée as generically as possible. We only use semantic loop unrolling until a defined loop bound to reduce the over-approximation in the analysis for all benchmarks.

*Blackbox Fuzzing with Blossom* We implement our novel blackbox fuzzing for kernel range computation in a tool we call Blossom. Blossom works by instrumenting the program to be analyzed. Blossom is implemented as an LLVM pass and works on C, C++, and Rust input programs with complex programming constructs and data types (and would work for any programming language that compiles to LLVM). Blossom takes as input the program $\mathcal{P}$, a configuration file that specifies the ranges of program inputs, the fuzzing technique that we want to execute (standard or guided blackbox), and a timeout. The LLVM pass automatically instruments $\mathcal{P}$ by inserting code that performs the indicated fuzzing process until the specified timeout, and records the ranges of kernel inputs.

In order to perform vanilla blackbox fuzzing, the code is instrumented with an `input generator` that utilizes the `srand()` function with distinctive seeds to randomly generate values of program inputs from the set of input bounds $\mathcal{I}$. This process is continued until the specified timeout.

*Guided Blackbox Fuzzing with Blossom* Algorithm 1 shows our guided blackbox fuzzing algorithm for generating program inputs to maximize kernel ranges. The algorithm is also implemented via LLVM-pass instrumentation in Blossom.

The inputs to Algorithm 1 are the program $\mathcal{P}$ with an identified set of kernels $\mathcal{K}$, a set of $n$ program input ranges ($\mathcal{I}$), and a timeout ($T$). The algorithm is also parameterized by the number of mutations $m$ and a constant $c$ that determines the neighborhood radii for all program inputs from which mutants (new program inputs) are drawn. The algorithm returns a set of kernel ranges $[\{\mathcal{R}_{lo}\}, \{\mathcal{R}_{hi}\}]$ (line 16). The goal is to compute the interval $[\{\mathcal{R}_{lo}\}, \{\mathcal{R}_{hi}\}]$ as wide as possible.

The algorithm keeps an input queue $Q$, which stores program inputs on which the program is to be executed. If $Q$ is empty, $m$ new random inputs taken from the program input ranges $\mathcal{I}$ are added to it (line 6–7). If $Q$ is not empty, the algorithm first dequeues one valuation of all the program inputs $\{v_1, \cdots, v_n\}$ from $Q$ (line 9), and executes the program $\mathcal{P}$ on these program inputs. During the execution of the program, the kernel monitor checks the kernel inputs and updates the kernel ranges as it is done in vanilla blackbox fuzzing (line 10). If the

**Algorithm 1** Guided Blackbox Fuzzing

1: **procedure** GUIDED-BLACKBOX($\mathcal{P}$, $\mathcal{I}$, $\mathcal{K}$, $T$, $m$, $c$)
2: $Q \leftarrow \phi$, $\{\mathcal{R}_{lo}\}$ =$\{$`DBL_MAX`$\}$, $\{\mathcal{R}_{hi}\}$ =$\{$`DBL_MIN`$\}$
3: $\{r_1, \cdots, r_n\} \leftarrow$ `computeRadii`$(\mathcal{I}, c)$ ▷ generates mutation radii
4: **while** $T \neq 0$ **do**
5: **if** $Q == \phi$ **then**
6: **for** $i$ from 1 to $m$ **do**
7: $Q \leftarrow$ `enqueue`(`generateRandomInput`$(\mathcal{I})$) ▷ generates random inputs
8: **else**
9: $\{v_1, \cdots, v_n\} \leftarrow$ `dequeue`$(Q)$
10: $[\{\mathcal{R}_{lo}\}, \{\mathcal{R}_{hi}\}] \leftarrow$ `executeAndmonitorKernels`$(\mathcal{K})$
11: **if** (`kernelRangeUpdated`$([\{\mathcal{R}_{lo}\}, \{\mathcal{R}_{hi}\}])$) **then**
12: **for** $i$ from 1 to $m-1$ **do**
13: $\{d_1, \cdots, d_n\} \leftarrow$ `mutate`$(v_1 \mp r_1, \cdots, v_n \mp r_n)$
14: $Q \leftarrow$ `enqueue`$(\{d_1, \cdots, d_n\})$
15: $Q \leftarrow$ `enqueue`(`generateRandomInput`$(\mathcal{I})$) ▷ avoids local max/min
16: **return** $[\{\mathcal{R}_{lo}\}, \{\mathcal{R}_{hi}\}]$ ▷ returns kernel input ranges kernel ranges were updated, i.e. we found an input that led to the kernel input being outside of the currently known range, we generate $m-1$ mutants from a program input $\{v_1, \cdots, v_n\}$ by randomly drawing inputs from its neighborhood $v_1 \mp r_1, \cdots, v_n \mp r_n$ and add them to the queue (line 12–14). (We draw mutants randomly from the neighborhood to reduce the possibility of duplicate program inputs.) The neighborhood, i.e. maximal distance of a mutant to the original program input, is defined by the neighborhood radii $\{r_1, \cdots, r_n\}$ (computed once on line 3) that depend on the width of each input range. Effectively, if an input range is large, then we will draw mutants from a larger neighborhood as well. This step enables to search in the neighborhood of the inputs that enlarged the ranges of the kernels recently. Then, we generate one random input for all variables in the whole input range (line 15). This step ensures that we do not get stuck in a local maximum or minimum. The whole process is repeated until timeout $T$.

## 4 Second Phase: Static Analysis with Daisy and CBMC

Next, we use the computed kernel ranges $\mathcal{R}$ as kernel input specifications (preconditions) and adapt the state-of-the-art roundoff-error analyzer Daisy [30] to verify the absence of cancellation errors and special float values. The translation of kernels and the precondition annotation to Daisy's input language in Scala is currently done manually, but could be automated in the future.

Daisy's core roundoff-error analysis performs a forward dataflow analysis. It computes ranges and worst-case absolute error bounds for each intermediate arithmetic (abstract syntax tree) expression using the interval and affine arithmetic abstract domains. As part of this analysis, it checks for overflows and invalid expressions that could lead to NaN values, as their absence is a prerequisite for a meaningful roundoff-error computation.

We extend Daisy to check at every intermediate expression for a possible cancellation, using the ranges and absolute error bounds that Daisy computes by default. At each binary arithmetic operation, we compare the relative errors of the operands with the relative error of the binary operation result. If the relative error increases more than a given factor, we report an error. We compute the relative error for an intermediate expression $x$ as the ratio of its worst-case absolute error bound divided by the smallest value that the range of $x$ contains. When the range of $x$ ($[x]$) contains zero, we divide instead by some small constant $c$, $\frac{\Delta_x}{\max(c, \min([x]))}$, to make relative errors always well-defined. While this does not compute a sound bound on the relative error, this is not needed for our purpose, since we are only interested in a relative comparison.

With this extension, we can prove for each kernel and the specified kernel input ranges, that cancellation and special values do not occur (but we cannot prove their presence). When Daisy cannot show this, it issues a warning with the possibly problematic intermediate expression. Spurious warnings for special values can be checked with a tool that performs exact reasoning, e.g. CBMC [52], and which reports a counterexample trace to the user who can use this trace to confirm whether the warning is genuine and if so, for debugging.

*Optimizing the Kernels* Daisy furthermore provides a rewriting optimization that finds an ordering of an arithmetic expression for which it can show a smaller (absolute) roundoff error [32]. The rewriting relies on the fact that floating-point arithmetic is not associative and distributive and hence different evaluation orders commit errors of different magnitudes. Daisy's algorithm uses real-valued identities such as associativity and distributivity to rewrite the expression. Using this optimization, we can thus locally improve the accuracy of the numerical kernels.

## 5 State of the Art on Real-World Programs

We collected a new set of real-world numerical programs from different application domains, as existing floating-point benchmark sets [29] cover kernels only. We first report on our experiments using existing representative state-of-the-art tools on these benchmarks, before evaluating our approach in Section 6.

*Benchmarks* All our benchmark programs are existing programs collected online from a variety of domains such as scientific computing simulations (`nbody`, `pendulum`, `lulesh`, `reactor`, `molecular`), physics algorithms (`fbench`, `arclength`), numerical methods (`linpack`) and machine learning (`linearSVC`). Table 1 provides an overview of the size and complexity of our benchmarks, as well as the number and arithmetic complexity of the kernels that we chose for verification. We also count the number of trigonometric operations (implemented in library functions) in the kernels, and the 'depth' column shows the number of function calls needed to reach the kernels from program entry.

| benchmark | lang. | LOC | #in. | #func. | #loops | kernels # | #arith op. | #trig. op. | depth |
|---|---|---|---|---|---|---|---|---|---|
| `arclength` [68] | C | 31 | 1 | 1 | 2 | 1 | 20 | 5 | 1 |
| `linearSVC` [8] | C | 32 | 4 | 1 | 3 | 1 | 7 | - | 1 |
| `raycasting` [6] | C | 94 | 2 | 4 | 3 | 1 | 4 | - | 4 |
| `nbody` [5] | C | 108 | 21 | 10 | 9 | 2 | 9, 22 | -,- | 2, 2 |
| `pendulum` [2] | C | 141 | 4 | 11 | 8 | 2 | 24, 42 | 2,11 | 4, 2 |
| `fbenchV2` [1] | C | 215 | 8 | 2 | 5 | 2 | 6, 14 | -,5 | 2, 2 |
| `molecular` [4] | C++ | 323 | 3 | 8 | 13 | 3 | 8, 12, 11 | -,-,3 | 1,1, 1 |
| `fbenchV1` [1] | C | 380 | 8 | 10 | 8 | 4 | 19, 6, 14, 36 | -,-,5,- | 5, 2, 2, 3 |
| `reactor` [7] | C++ | 467 | 4 | 11 | 2 | 3 | 14, 11, 13 | -,2,2 | 2, 0, 1 |
| `linpack` [3] | C | 544 | 5 | 12 | 31 | 1 | 8 | - | 2 |
| `lulesh` [51] | C++ | 2187 | 5 | 43 | 74 | 4 | 109, 77, 14, 41 | -,-,-,- | 6, 7, 6, 7 |

**Table 1.** Benchmark statistics

These benchmarks are single-threaded C or C++ floating-point programs with arrays, structures, branching, loops, and function calls (we translated the `pendulum` benchmark manually from Python to C). We modified the benchmarks by replacing dynamic memory allocation, pointer arithmetic, and I/O operations as appropriate, since these are challenging for most program analyses. We considered two versions of `fbench`: one with user-defined trigonometric functions (V1) and 380 LOC, and another with their library versions (V2). We specified bounds on the program inputs manually and identified a set of numerical kernels containing a large number of arithmetic operations.

*State of the Art* We first evaluate existing state-of-the-art tools on our benchmark set. For this, we choose CBMC, Astrée and AFLGo as representatives for model checking, abstract interpretation and directed greybox fuzzing, respectively. To the best of our knowledge, AFLGo was not used for floating-point debugging before. These tools check for assertion violations, so we have added assertions to our chosen kernels to check for absence of Infinity and NaN using the standard library functions `isinf` and `isnan`.

We do not include a deductive verifier (e.g. [24]) in this comparison, because it requires detailed user annotations of every function. None of the state-of-the-art static roundoff-error analysis tools [43,33,31,30,60,65,72] work on the whole applications in our benchmark set. Available dynamic analyses for finding large roundoff errors [10,21,77,21,78,44] or special values [38,57,9] also work only on smaller programs (often restricted to kernels). Only the dynamic-analysis tool FPDebug [10] has been shown to scale beyond numerical kernels, but unfortunately the code has not been actively maintained over the years.

All experiments are done for 64-bit precision and on a Debian server system with 2.67GHz and 50GB RAM. We have used CBMC version 5.12 with MiniSat 2.2.0 (we have observed in our preliminary experiments that CBMC performs better with MiniSat), Astrée's `linux64_b5162300_release` and AFLGo downloaded on June 9, 2020. We have set a 1-hour time budget for all experiments and unrolled all loops for 50 iterations for both CBMC and Astrée.

With CBMC and Astrée, we are able to prove the absence of special float values in `linearSVC` and `rayCasting`, two of the smallest benchmarks. Additionally, Astrée also proves the absence of special values in kernels 1 and 5 in `fbenchV1`. For all other C benchmarks (Astrée does not work on C++ programs), Astrée generates warnings for the potential existence of special values. With AFLGo, however, we do not find any special values within the time limit.

For the `nbody` and `pendulum` benchmarks, we originally had larger program input ranges. For these, AFLGo was able to show the presence of special values in the kernels, suggesting that greybox fuzzing is effective for detecting special values. For the subsequent experiments, we have used tighter program input ranges to avoid special values.

## 6 Evaluation of our Two-Phase Approach

We next evaluate our two-phase approach. For a fair comparison with the state-of-the-art tools, we designate a 1-hour time limit for the entire analysis, allocating 50 minutes for generating the kernel ranges and 10 minutes for the kernel analysis. We have empirically evaluated the effect of the time limit and observed that increasing the time does not affect the results of our benchmarks, but a smaller time limit led to worse results.

*Computing Kernel Ranges* The main step is the computation of the kernel ranges. We compare the kernel ranges obtained with blackbox fuzzing (BB), guided blackbox fuzzing (GBB) (both implemented in Blossom), AFLGo with our iterative widening (AFLGo), and a combination of BB and AFLGo iterative widening (BB+AFLGo). We have empirically determined that with 5 mutants GBB performs the best for all our benchmarks. For AFLGo, we first fuzz the program for 5 minutes and then run our iterative widening that employs the fuzzer in a refinement loop to widen the so-obtained ranges (see Section 2.1) for the next 45 minutes. For BB+AFLGo, we use Blossom's blackbox fuzzing for 25 minutes to generate the initial ranges. On these ranges, we use our range-widening technique with AFLGo for the next 25 minutes.

To compare the obtained kernel ranges, we first compute the width of each kernel range ($\overline{x} - \underline{x}$) and show in Table 2 the average width over all kernel inputs and over 5 runs with different random seeds. For our dynamic analyses, we want to maximize the kernel ranges to cover as many kernel inputs as possible.

We also add the sound over-approximated ranges computed by Astrée, whenever these are available. While Astrée produces a warning *inside* the `arclength` kernel, it still computes a finite range for the kernel *input*.

In 5 out of the 7 kernels where Astrée finds non-trivial ranges, our fuzzing techniques also compute ranges that are close to Astrée's. They are even equal in the case of `rayCasting`. In the other 2 cases, Astrée reports big ranges whereas

| benchmark | kernel | #vars | Astrée | BB | AFLGo | BB+AFLGo | GBB | kernel analysis |
|---|---|---|---|---|---|---|---|---|
| | | | avg range width | | | | | |
| `arclength` | 1 | 1 | 6.16e+4 | **3.14** | **3.14** | **3.14** | **3.14** | ✓ |
| `linearSVC` | 1 | 4 | 3.73 | **3.73** | 3.71 | 3.72 | **3.73** | (✓) |
| `rayCasting` | 1 | 5 | 12.20 | **12.20** | **12.20** | **12.20** | **12.20** | ✓ |
| `nbody` | 1 | 6 | ∞ | 1.09e+5 | 6.67e+4 | 1.21e+5 | **1.02e+8** | ✓ |
| | 2 | 9 | ∞ | 1.25e+4 | 8.45e+3 | 1.19e+4 | **8.91e+6** | ✗ |
| `pendulum` | 1 | 4 | ∞ | 14.80 | 12.86 | **14.82** | 14.56 | ✓ |
| | 2 | 5 | ∞ | 22.38 | 17.61 | **22.39** | 22.16 | ✓ |
| `fbenchV2` | 1 | 5 | 24.60 | **20.46** | **20.46** | **20.46** | **20.46** | ✗ |
| | 2 | 5 | ∞ | **21.36** | **21.36** | **21.36** | **21.36** | ✗ |
| `fbenchV1` | 1 | 1 | 403.00 | **0.18** | **0.18** | **0.18** | **0.18** | ✓ |
| | 2 | 5 | 20.50 | **20.46** | **20.46** | **20.46** | **20.46** | ✗ |
| | 3 | 5 | ∞ | 21.36 | 21.36 | **24.76** | 21.36 | ✗ |
| | 4 | 1 | 1.57 | **1.54** | **1.54** | **1.54** | **1.54** | ✓ |
| `linpack` | 1 | 8 | ∞ | 3.60e+6 | 4.44e+3 | 3.60e+6 | **2.11e+269** | ✗ |
| `molecular` | 1 | 4 | ✗ | **9.04** | **9.04** | **9.04** | **9.04** | ✗ |
| | 2 | 6 | ✗ | **1.86** | **1.86** | **1.86** | **1.86** | ✓ |
| | 3 | 7 | ✗ | **12.88** | **12.88** | **12.88** | **12.88** | ✓ |
| `reactor` | 1 | 1 | ✗ | **1.00** | **1.00** | **1.00** | **1.00** | ✓ |
| | 2 | 6 | ✗ | 1.43e+2 | 9.35e+1 | 1.43e+2 | **1.46e+2** | ✗ |
| | 3 | 1 | ✗ | **2.50** | **2.50** | **2.50** | **2.50** | ✓ |
| `lulesh` | 1 | 24 | ✗ | **4.97** | 4.80 | **4.97** | 4.95 | (✓) |
| | 2 | 18 | ✗ | **6.09** | 5.51 | 5.50 | 5.89 | ✓ |
| | 3 | 9 | ✗ | **3.48** | 3.09 | 3.42 | 3.25 | ✓ |
| | 4 | 12 | ✗ | **5.95** | 5.49 | 5.93 | 5.77 | ✓ |

**Table 2.** Comparison of kernel ranges generated by different techniques and settings all fuzzing techniques compute smaller ranges with the same width, suggesting a possible large over-approximation of Astrée's ranges (or the inability of fuzzers to discover new kernel inputs within the time limit).

In the other cases, when Astrée finds unbounded ranges or does not work, we observe that for all but 3 kernels, all four fuzzing techniques compute very similar range widths. For 3 kernels, however, GBB finds significantly larger ranges, thus discovering kernel inputs that the other methods are not able to find. We thus conclude that guided blackbox fuzzing appears to be most suitable for computing kernel ranges in our benchmarks, as it can discover apparent outliers.

AFLGo often computes the smallest ranges. Our hypothesis is that because AFLGo aims to maximize the number of paths in the program to reach the target locations in the kernels, it focuses on generating values to find new paths rather than generating values exercising an already found path that may increase the width of the kernel ranges.

| benchmark | kernel | #vars | BB | AFLGo | BB+AFLGo | GBB |
|---|---|---|---|---|---|---|
| `linearSVC` | 1 | 4 | - | 2.21 | - | - |
| `nbody` | 1 | 6 | 121.05 | 312.93 | 144.86 | 181.26 |
| | 2 | 9 | 155.31 | 226.10 | 127.25 | 206.20 |
| `pendulum` | 1 | 4 | 0.69 | 51.77 | 0.57 | 5.25 |
| | 2 | 5 | 0.69 | 44.37 | 0.54 | 4.48 |
| `fbenchV2` | 1 | 5 | - | - | 1.99 | - |
| | 2 | 5 | - | 0.04 | - | - |
| `fbenchV1` | 1 | 1 | - | 0.03 | - | - |
| | 2 | 5 | - | - | 1.99 | - |
| | 3 | 5 | - | 0.04 | 8.85 | - |
| `linpack` | 1 | 8 | 0.01 | 100.15 | - | 114.58 |
| `molecular` | 2 | 6 | 0.25 | 8.0 | 0.15 | 0.33 |
| `reactor` | 1 | 1 | - | 0.01 | - | - |
| | 2 | 6 | 2.51 | 11.32 | 2.91 | 2.80 |
| | 3 | 1 | - | 0.01 | - | - |
| `lulesh` | 1 | 24 | 1.67 | 6.76 | 1.74 | 2.50 |
| | 2 | 18 | 4.28 | 19.73 | 15.59 | 6.96 |
| | 3 | 9 | 7.14 | 23.25 | 10.55 | 11.97 |
| | 4 | 12 | 3.91 | 16.13 | 3.49 | 5.88 |

**Table 3.** Variation of computed kernel range widths (from the average width) for our three fuzzing techniques (in %), '-' denotes no variation

*Effect of Randomness* All fuzzing techniques (BB, GBB, AFLGo) rely on randomness. To evaluate how the computed kernel ranges are affected by it, we calculate the variation of the range widths compared to the average range width (per variable) over 5 runs. For 7 kernels, we do not detect any variation at all for any of the methods; Table 3 shows the variations for the remaining kernels.

We observe that all methods have large variations for the benchmarks `nbody` and `linpack`, i.e. those for which GBB has found very large ranges. This suggests that there are a few corner-case inputs that lead to large kernel ranges (which only GBB was able to reliably find). Further, we see that AFLGo has a large range variation due to randomness for a few additional benchmarks, whereas BB and GBB have variations that are relatively small.

*Conditional Kernel Verification* We were able to (conditionally) prove the absence of special floating-point values for 16 out of the 24 kernels, and (conditionally) prove the absence of cancellation errors for 14 of those kernels. We show these results in the last column of Table 2: '✓' indicates that Daisy could prove both the absence of special values and cancellation in the kernel for the specified kernel ranges, '(✓)' indicates that only the absence of special values could be verified, and '✗' shows when Daisy reports a special-value warning. For the relatively small benchmarks arclength, linearSVC and rayCasting, our verification of the kernels is sound, i.e. unconditional, as we used ranges computed by Astrée.

When Daisy reports a warning, it is not guaranteed that a kernel can actually compute a special-value result, because of 1) Daisy's over-approximation of the concrete program semantics, and because 2) the range we compute may contain values that are not feasible in the actual program execution. To help developers debug warnings reported by the static analyzer, we use CBMC on those kernels.

CBMC reports counterexamples in all kernels for which Daisy reports warnings. Upon code inspection, however, we identified the counterexamples of nbody and fbench to be spurious for the particular program inputs we consider. In these cases, the true kernel input range was discontinuous, and the counterexamples were reported for the infeasible inputs. In particular, in kernel 2 of nbody, a NaN could be produced if the two bodies that are simulated collide, which would not happen for the initial conditions that we chose. Similarly, the kernels in the ray-tracing algorithm of fbench could produce Infinity, if the ray was chosen in a very particular way. With the program input ranges we have chosen, this was impossible.

For linpack, the arithmetic overflow reported is indeed genuine, since a division by zero can occur before the kernel if the input matrix contains a zero on the diagonal, which leads to undefined behavior and the huge range of the kernel inputs. Similarly, for molecular and reactor, arithmetic overflow can occur for a specific position of molecules and a specific value of the angle between particle's direction and the X-axis, respectively.

We note that given the counterexamples produced by CBMC, we could straight-forwardly identify the warnings as spurious or genuine. In future work, one could consider refining the kernel monitoring, such that it would not only track a single range per kernel but could detect discontinuous ranges.

Our extension of Daisy reports cancellation-error warnings for one kernel of linearSVC and one kernel of lulesh. We have used a threshold of $10^3$ for reporting cancellation, i.e. if the relative errors of the operands and the result differ by more than three orders of magnitude, we report an error. We inspected the kernel code and confirmed that the cancellation warnings are genuine, i.e. there are indeed inputs that will result in a large roundoff error. The number of cancellations found may seem small. We suspect that this is the case, because our benchmarks were mostly written as reference or example programs (e.g. lulesh was developed to be a representative hydrodynamics simulation code), hence we expect them to be carefully developed and tested.

*Kernel Optimization* We have additionally applied Daisy's rewriting optimization on those kernels for which Daisy does not report possible special values. With this procedure, we could reduce the roundoff errors in 8 of the kernels out of which 6 cases are notable. We could reduce the error by 9.5% for linearSVC, 7.1% and 3.3% for two outputs of kernel 2 in pendulum, by 19.8%, 4.0%, 5.8%, and 5.8% for different kernel outputs of lulesh, and by 33.3% for one output of molecular. From these experimental results, we conclude that the ranges that we inferred in the first phase are actually useful for kernel analysis.

## 7 Related Work

Abstract interpretation-based techniques are in principle uniquely suitable for verifying the absence of special values and safety in floating-point programs. We have chosen Astrée [63] in this work because it is an industrial-strength tool, and as such, supports a wide range of C programs and is designed for scalability. Apron [50] is a library of numerical abstract domains that are sound w.r.t. floating-point arithmetic, and includes, for instance, the domain of polyhedra [19], which is, however, significantly more expensive than the interval arithmetic domain that we use. ELINA [71] provides performance-optimized implementations of many numerical abstract domains, but its polyhedra domain does not support floating-point arithmetic.

These domains only bound variable values; abstract domains [43,33,31,30] or optimization-based static analyses [60,65,72] for bounding roundoff errors provide nontrivial results only for relatively small kernels. For the second step in our framework, we could have in principle chosen any of these tools; we chose Daisy because we found it easy to modify for our needs, and because it already includes the rewriting optimization.

In the space of deductive verification, besides Frama-C [24], the Boogie intermediate verification language [53] also has support for floating-point arithmetic and discharges the verification conditions using the Z3 SMT solver. Similarly, bounded model checking [52] is limited by the performance of the underlying SAT/SMT solvers. While the floating-point support in today's SMT solvers [17,16] has improved significantly in recent years, it is still limited to relatively few arithmetic expressions.

Many interactive theorem provers have floating-point formalizations [49,15,37]. While these do allow to prove complex functional properties [13,14,46], the proofs are largely manual and require significant expertise.

Blackbox testing has been explored to find large roundoff errors by executing a higher-precision version of the program side-by-side [10,21,77]. Recently, whitebox testing has been used for detecting overflows [38], by phrasing the search as a mathematical optimization problem, and large roundoff errors [21,78], by adapting the notion of condition numbers. KLEE-Float [57], FPGen [44] and Ariadne [9] use symbolic execution for finding bugs in floating-point code, including overflows and large precision loss and cancellation. While KLEE-Float relies on the floating-point SMT decision procedures, Ariadne approximates the path constraints and uses the real-valued theory. FPGen injects specialized inaccuracy checks to find cancellations. Only FPDebug [10] has been shown to scale beyond numerical kernels and, to the best of our knowledge, none of the dynamic techniques have been used to obtain range information.

Once a large roundoff error has been identified, Herbgrind [69] can help to locate its root cause, which may be in a different instruction than where the error becomes significant. Herbgrind is thus complementary to our work and may be used to locate root causes of potential cancellation errors reported by Daisy.

Rewriting floating-point expressions in order to optimize roundoff errors has been explored in the tool Herbie [67] and others [74,76]. These approaches attempt to repair unstable code, checking accuracy using a dynamic analysis. They are alternatives to using Daisy for the second step in our framework. Alternative program optimizations that we have not explored in this work, but that also require range information, include mixed-precision tuning [32,20,68] and general non-semantics preserving approximation [70].

Apart from AFLGo [12], there is a wide range of targeted greybox fuzzers, such as those targeting specified program locations [18], rare branches [54], unexplored branches [55,73], or potential vulnerabilities [39,45,22,56]. In our setting, we require fuzzers like AFLGo to target the specific program locations of kernels.

There is a significant body of work on guiding program analyzers. In particular, test case generation is typically guided by a static analysis toward specific parts of the code (e.g., [27,35,66,41,40,58,62,28,59,23,36,34,75,44]). Our approach is similar to these techniques as it infers input ranges to guide verifiers of numerical kernels toward those kernel executions that are relevant in the context of the containing application.

## 8 Conclusion

Even though floating-point programs have received a lot of attention recently, their focus has been largely on verifying or debugging arithmetic kernels. Our review of existing techniques and tools has shown that few approaches with specific floating-point support are applicable to whole programs without significant user expertise. We have found, however, that standard greybox fuzzing proved to be effective in detecting overflows and NaNs. Meanwhile, static-analysis techniques to show the absence of special values and cancellation errors remain limited to programs with few bounded loops and numerical kernels, respectively.

Instead of trying to scale up existing roundoff-error analysis tools to whole programs, we *combine* them with more scalable analyses that compute the kernel preconditions needed for the roundoff analyses to work. We showed how relatively small adaptations to well-known techniques of directed blackbox and greybox fuzzing are enough to realize such a framework. Together with modifications to an existing roundoff-error analyzer, we are able to *conditionally verify* the absence of special values and cancellations in a number of numerical kernels in realistic floating-point programs that are out of reach for today's analyses. At the same time, our analysis is precise enough to identify several cases of cancellations. While our approach is not suitable and not intended for certification of safety-critical systems, we believe that it nonetheless provides valuable debugging feedback for many real-world applications.

## Acknowledgements

This research was partially funded by the Deutsche Forschungsgemeinschaft (DFG, German Research Foundation) project 387674182 and project 389792660 as part of TRR 248 (see `https://perspicuous-computing.science`). We also thank Dr.-Ing. Jörg Herter from AbsInt for the training and assistance with Astrée.
